# Resorcine and Its Employment in Therapeutics

**Published:** 1882-06

**Authors:** 


### RESORCINE AND ITS EMPLOYMENT IN THERAPEUTICS.

MM. Dujardin-Beaumetz and Callias have made an extended study of this substance and its use, in the *Bull. Gen. de Therapeutique.*

This substance was discovered by two Viennese chemists, Hlassiwetz and Barth, in 1860, among the products of fusion obtained by treating galbanum with potash; later on it was obtained from gum ammoniac and assafœtida.

Resorcine is very soluble in water, has a feeble odor, somewhat resembling that of carbolic and benzoic acid, and has the same properties as carbolic acid; a one per cent. solution acting as an anti-ferment, and a one and one-half per cent. solution as an antiputrid.

The authors are of opinion that this antiseptic, on account of its extreme solubility, its very feeble odor, its toxic effects being slight as compared with carbolic acid, it being also much less caustic, should be preferred to this latter in surgical practice.

Gray's Anatomy has been translated into the Chinese language, and published in six volumes, at Foochow.

